# Niemann-Pick disease type C symptomatology: an expert-based clinical description

**DOI:** 10.1186/1750-1172-8-166

**Published:** 2013-10-17

**Authors:** Eugen Mengel, Hans-Hermann Klünemann, Charles M Lourenço, Christian J Hendriksz, Frédéric Sedel, Mark Walterfang, Stefan A Kolb

**Affiliations:** 1 Department of Lysosomal Storage Disorder, Villa Metabolica, Center for Paediatric and Adolescent Medicine, University Medical Center of the Johannes Gutenberg University Mainz, Langenbeckstrasse 1, 55131 Mainz, Germany; 2 Department of Psychiatry, University of Regensburg, 93053 Regensburg, Germany; 3 Medical Genetics Service, Clinics Hospital of Ribeirão Preto, University of São Paulo, São Paulo, Brazil; 4 Manchester Academic Health Science Centre (MAHSC), University of Manchester, Salford Royal Hospital NHS Foundation Trust, Stott Lane, Manchester M6 8HD UK; 5 Department of Neurology and Reference Center for Lysosomal Diseases, Groupe Hospitalier Pitié-Salpêtrière, 75013 Paris, France; 6 Department of Neuropsychiatry, Royal Melbourne Hospital and Melbourne Neuropsychiatry Center, University of Melbourne, 3050 Melbourne, Australia; 7 Actelion Pharmaceuticals Ltd, 4123 Allschwil, Switzerland

**Keywords:** Niemann-Pick disease type C, Lysosomal lipid storage disease, Splenomegaly, Ataxia, Dystonia, Vertical supranuclear gaze palsy, Gelastic cataplexy, Cognitive impairment, Diagnosis

## Abstract

Niemann-Pick disease type C (NP-C) is a rare, progressive, irreversible disease leading to disabling neurological manifestations and premature death. The estimated disease incidence is 1:120,000 live births, but this likely represents an underestimate, as the disease may be under-diagnosed due to its highly heterogeneous presentation. NP-C is characterised by visceral, neurological and psychiatric manifestations that are not specific to the disease and that can be found in other conditions. The aim of this review is to provide non-specialists with an expert-based, detailed description of NP-C signs and symptoms, including how they present in patients and how they can be assessed. Early disease detection should rely on seeking a combination of signs and symptoms, rather than isolated findings. Examples of combinations which are strongly suggestive of NP-C include: splenomegaly and vertical supranuclear gaze palsy (VSGP); splenomegaly and clumsiness; splenomegaly and schizophrenia-like psychosis; psychotic symptoms and cognitive decline; and ataxia with dystonia, dysarthria/dysphagia and cognitive decline. VSGP is a hallmark of NP-C and becomes highly specific of the disease when it occurs in combination with other manifestations (e.g. splenomegaly, ataxia). In young infants (<2 years), abnormal saccades may first manifest as slowing and shortening of upward saccades, long before gaze palsy onset. While visceral manifestations tend to predominate during the perinatal and infantile period (2 months–6 years of age), neurological and psychiatric involvement is more prominent during the juvenile/adult period (>6 years of age). Psychosis in NP-C is atypical and variably responsive to treatment. Progressive cognitive decline, which always occurs in patients with NP-C, manifests as memory and executive impairment in juvenile/adult patients. Disease prognosis mainly correlates with the age at onset of the neurological signs, with early-onset forms progressing faster. Therefore, a detailed and descriptive picture of NP-C signs and symptoms may help improve disease detection and early diagnosis, so that therapy with miglustat (Zavesca®), the only available treatment approved to date, can be started as soon as neurological symptoms appear, in order to slow disease progression.

## Introduction

Niemann-Pick disease type C (NP-C) is a rare, progressive genetic lysosomal lipid storage disease caused by mutations in the *NPC1* or *NPC2* gene [1,2]. It is a highly heterogeneous disease, characterised by visceral, neurological and psychiatric manifestations that can present alone, or in specific or non-specific combinations. More-over, age at onset and disease course vary greatly from one patient to another, including among siblings [1,2]. Patients often first present to general practitioners; due to its challenging presentation, especially for non-specialists, the disease often remains undetected for many years, with an average delay in diagnosis of 5–6 years from onset of neurological symptoms [3-6]). Early diagnosis is essential so that therapy with miglustat (Zavesca®, Actelion Pharmaceuticals Ltd, Allschwil, Switzerland), the only available disease-specific therapy approved for NP-C [7], can be initiated as soon as neurological symptoms appear in order to slow the progression of neurological damage.

Individual NP-C manifestations are not specific to the disease, but the combination of multiple signs and symptoms shows more diagnostic specificity for NP-C, which may aid with disease detection. Therefore, understanding how and in which combination these manifestations present in the context of NP-C may help physicians identify possible suspected cases of NP-C.

This review provides an expert-based descriptive clinical picture of NP-C that goes beyond the scope of currently available information to practising clinicians, and includes details on specific signs and symptoms and how they present in individuals with NP-C. This qualitative description of NP-C signs and symptoms is not limited to published clinical study data, but also reflects experts’ opinion drawn from clinical practice and personal experience. It aims to increase disease awareness among physicians in order to improve early diagnosis and timely referral to specialists of patients with suspected disease.

### Disease description, epidemiology and aetiology

NP-C is a genetic, progressive, irreversible and chronically debilitating neurovisceral lysosomal lipid storage disease leading to premature death [1,2]. NP-C is generally panethnic, although some mutations may occur with higher incidence in defined ethnic groups [8,9]. The minimal estimated incidence of the disease is one case in every 120,000 live births [2], although this value is likely to represent an underestimation due to failure to reliably recognise the disease (see below).

NP-C is a genetic autosomal recessive disease caused by mutations in the genes *NPC1* (~95% of cases), *NPC2* (~4% of cases) and possibly other as yet unidentified genes (~1% of cases) [1,10,11]. As of November 2012, 252 gene sequence variants have been listed for *NPC1* and 18 for *NPC2*, with a majority of point mutations [12]. Mutations in either gene lead to the same cellular deficits, including impaired cholesterol esterification [13] and intracellular lipid trafficking [14]. This results in intracellular accumulation of different lipids and altered sphingolipid metabolism leading to the pathophysiology of the disease. Putative functions of NPC1 and NPC2 and their role in the pathophysiology of NP-C have been described more comprehensively elsewhere [2,14,15]. Depending on whether the *NPC1* or *NPC2* gene carries mutations, the disease is sometimes referred to as NP-C1 or NP-C2, respectively. For certain gene mutations, there appears to be a correlation between genotype and the severity of the neurological course of the disease [16]. For a comprehensive definition of the disease, including its historical delineation, we refer the reader to a recent review [2].

### Clinical description and differential diagnosis

NP-C is a complex disease that first of all affects the spleen, liver and brain, resulting in visceral abnormalities as well as neurological and psychiatric manifestations (Table 1). The combined presentation of visceral, neurological and psychiatric manifestations should therefore lead to the consideration of NP-C in the differential diagnosis of this symptomatology. Examples of combinations which are strongly suggestive of NP-C include: splenomegaly and vertical supranuclear gaze palsy (VSGP); splenomegaly and clumsiness; splenomegaly and schizophrenia-like psychosis; psychotic symptoms and cognitive decline; and ataxia with dystonia, dysarthria/dysphagia and cognitive decline [17] (Table 2).

**Table 1:** Classification of signs and symptoms in NP-C

**Visceral**	
	Isolated unexplained splenomegaly
	Hepatomegaly/Splenomegaly
	Prolonged neonatal cholestatic jaundice
	Hydrops foetalis or foetal ascites
	Pneumopathologies (aspiration pneumonia, alveolar lipidosis, interstitial lung involvement)
	Mild thrombocytopenia
**Neurological**	
	Vertical supranuclear gaze palsy
	Gelastic cataplexy
	Ataxia
	Dystonia
	Dysarthria
	Dysphagia
	Hypotonia
	Clumsiness
	Delayed developmental milestones
	Seizures
	Hearing loss
**Psychiatric**	
	Developmental delay and pre-senile cognitive decline
	Organic psychosis
	Disruptive/aggressive behaviour
	Progressive development of treatment-resistant psychiatric symptoms

*Abbreviation*: NP-C, Niemann-Pick disease type C.

**Table 2:** Sign and symptom combinations strongly suggestive of NP-C

Splenomegaly	+	Vertical supranuclear gaze palsy
		Hypotonia
		Schizophrenia-like psychosis
		Gelastic cataplexia
		Delayed developmental milestones
Ataxia	+	Dystonia
		Dysarthria/dysphagia
		Cognitive decline
Psychotic symptoms	+	Cognitive decline

The combination of one of the symptoms on the left with at least one of those on the right is strongly suggestive of NP-C.

*Abbreviation*: NP-C, Niemann-Pick disease type C.

The combination of cerebellar ataxia and dystonia of the hands and the face is one motor hallmark of NP-C. However, a combination of cerebellar ataxia and dystonia can also be found in other diseases, including mitochondrial disorders such as Leigh syndrome, GM2 gangliosidosis, ataxia with oculomotor apraxia type I, Gaucher disease type 3 (GD3), and spinocerebellar ataxia. Therefore, even if this combination is highly suggestive of NP-C, it does not necessarily confirm a diagnosis of NP-C.

NP-C presentations are often categorised based on the age at onset of the neurological symptoms: early-infantile (2 months–2 years of age), late-infantile (2–6 years of age), juvenile (6–12 years of age), adolescent/adult (>12 years of age). The perinatal form (up to the age of 2 months) is characterised by systemic symptoms only [1,2].

### Visceral symptoms in NP-C

#### Isolated unexplained splenomegaly with or without hepatomegaly

Historical or current isolated unexplained splenomegaly, with or without hepatomegaly, is observed in the majority of patients with NP-C [1] and is the strongest visceral indicator of the disease [18]. When present in combination with other neurological and/or psychiatric symptoms, including VSGP, ataxia and schizophrenia-like symptoms, isolated splenomegaly becomes highly suggestive of NP-C [1]. Isolated unexplained splenomegaly should always lead to the inclusion of NP-C in the differential diagnosis, and hence trigger a search for other symptoms of the disease. Splenomegaly in NP-C presents along a continuum, ranging from slight to tremendous enlargement, even in young children. Importantly, the degree of splenomegaly does not correlate with neurological manifestations, disease severity or illness stage. Absence of splenomegaly should not lead to the exclusion of NP-C.

In young patients, splenomegaly can be assessed by turning the patient on the right side in order to have the spleen falling downwards. In this position, the spleen should not be palpable under normal conditions. A palpable spleen indicates that its size is increased by at least two-fold. In adolescent and adult patients, mild splenomegaly may only be detected by abdominal imaging such as ultrasound [1,3].

Unlike splenomegaly, hepatomegaly is less frequently observed in adult patients with NP-C [1,3]. Hepatomegaly presentation in NP-C is non-specific; it generally appears at the same age as splenomegaly, or in some cases may present without it, which is often attributed to a failure to clinically detect splenomegaly in the absence of an abdominal ultrasound. Hepatomegaly can be detected by palpation of the patient lying in a supine position, starting from the right flank and slowly moving upwards. A palpable lower edge of the liver indicates hepatomegaly. Upward palpation should also be started from the left flank, as occasionally only the left lobe of the liver is enlarged, crossing over the midline. While up to a two-fold increase in spleen size can remain impalpable, liver enlargement as small as 1 cm can be readily felt. Notably, the degree of hepatomegaly and splenomegaly are not related, and unlike splenomegaly [2], hepatomegaly does not appear to resolve spontaneously.

Isolated spleno- or hepatosplenomegaly also occur in some other inherited metabolic diseases, such as mucopolysaccharidoses, glycogen storage disorders, Sandhoff disease, GD3, lysosomal acid lipase deficiency and Niemann-Pick disease type A and B [1].

#### Prolonged or unexplained neonatal cholestatic jaundice

Signs of perinatal liver involvement range from transient conjugated hyperbilirubinaemia to severe cholestatic hepatopathy leading to liver failure and death in the first year of life.

A history of prolonged or unexplained neonatal cholestatic jaundice is a strong visceral indicator of NP-C [18]. Generally defined as prolonged conjugated hyperbilirubinaemia that occurs in newborns, it is frequently observed in patients with early- and late-infantile disease onset [19-22]. In NP-C, jaundice always has a cholestatic origin and is defined by a conjugated bilirubin level >1.2 mg/dL and/or >30% of total bilirubin for a period of over 2 weeks [1].

Conjugated bilirubin levels and speed of symptom resolution are non-specific in NP-C. Acholic stools can be a characteristic of NP-C-related cholestatic jaundice. Since this condition does not require phototherapy (unlike unconjugated jaundice), its symptoms may often not be recalled by parents and hence may be missed when obtaining medical history.

Cholestasis should always lead to the consideration of NP-C in the differential diagnosis of neonatal jaundice. In neonates and young infants, NP-C should be differentiated from other causes of cholestatic jaundice, e.g. idiopathic neonatal hepatitis or biliary atresia [1]. The occurrence of isolated spleno- or hepatosplenomegaly is a helpful indicator and should raise suspicion of NP-C [18].

#### Hydrops foetalis or sibling with foetal ascites

The presence of perinatal hydrops foetalis or a sibling with foetal ascites occurs frequently in newborns with lysosomal storage diseases [23,24]. However, they are considered ancillary indicators of NP-C as they are less frequent in patients with this disease [18,20,25]. In NP-C, hydrops foetalis has a non-immune origin and always presents with ascites, never as a classical hydrops foetalis. It is usually detected upon antenatal foetal ultrasonography scanning and presents as global swelling, with some fluid accumulating in the abdominal cavity or around the heart. Hydrops foetalis may be missed during the medical and family history, because the information is often held with obstetricians rather than paediatricians. Differential diagnosis for hydrops foetalis include chromosomal disorders, congenital heart malformations, infectious diseases and haemoglobin disorders.

#### Other symptomatology

Lung disease can occur in both NP-C1 and NP-C2 disease, and is usually associated with more severe types of the disease. In NP-C2, the clinical picture can be similar to that of chronic lung disease of the newborn in the absence of a history to support it. Helical computed tomography imaging of the chest may occasionally show classical interstitial lung disease. These features have been poorly described in the literature [26-28] but are often anecdotally reported by experts. There is no specific therapy for the pulmonary manifestations, although bone marrow transplantation may offer some resolution in NP-C2 [29].

Mild thrombocytopenia in newborns or toddlers with NP-C has been anecdotally reported, with limited evidence ([1] and CJH, personal communication). This finding is non-specific, as platelet abnormalities are common in cases of splenomegaly and have been described in other lysosomal storage diseases. Bone marrow infiltration with foam cells may cause platelet abnormalities in newborns, although this remains to be confirmed. Foamy cells can be detected by bone marrow aspiration. Usually, patients with classical foamy cells are the most severely affected and present with large splenomegaly, low platelet counts, bone infiltrates, and a most widespread presentation. It should be noted, however, that patients may rarely present with classical foamy cells and splenomegaly from an early age, yet remain asymptomatic for the neurological manifestations of the disease.

### Neurological symptoms in NP-C

#### Vertical supranuclear gaze palsy

VSGP is characterised by impaired saccadic movements of the eyes in the vertical direction as a result of a supranuclear lesion [30]. Patients with VSGP exhibit a deficit in voluntary and reflexive vertical saccades, as well as in vestibulo-ocular nystagmus. Along with gelastic cataplexy, VSGP is the strongest neurological indicator of NP-C [18], and becomes highly predictive of NP-C when found in combination with other manifestations, such as splenomegaly, ataxia or psychosis. During later stages of the disease, horizontal saccades are also affected, reflecting progressive degeneration of neurons within the paramedian pontine reticular formation, which controls horizontal saccades [30-32]. Neuronal loss in the rostral interstitial nuclei of the medial longitudinal fasciculus results in a palsy of voluntary and reflexive vertical saccades, as well as of the quick phases of vertical nystagmus [1,30]. Importantly, the vestibulo-ocular reflex, which depends on the vestibular nuclei under the control of cerebellar projections, is often preserved until very late in the disease progression [1,3,32,33]. Slow and hypometric vertical saccades followed by compensating head movement may be the first sign in children and infants long before gaze palsy develops, and may start in infants below the age of 2 years. Children may fall as they become unable to adjust their vision for stairs or other obstacles. In cases where children are clumsy and fall often, but have not been diagnosed with VSGP, the initiation, velocity and amplitude of upward saccades should be assessed. Typically, children close their eyes when trying to look up and re-open them once they have reached their upward position; alternatively, they may blink when asked to look up. These are features that a physician should pay attention to when conducting a clinical examination. In older children, adolescents and adults, downward gaze appears to be affected first [3,30]; it can manifest as a tilt of the head for everyday tasks such as writing, driving, or using a cash machine, or as a difficulty when descending stairs [3].

**Figure 1:**
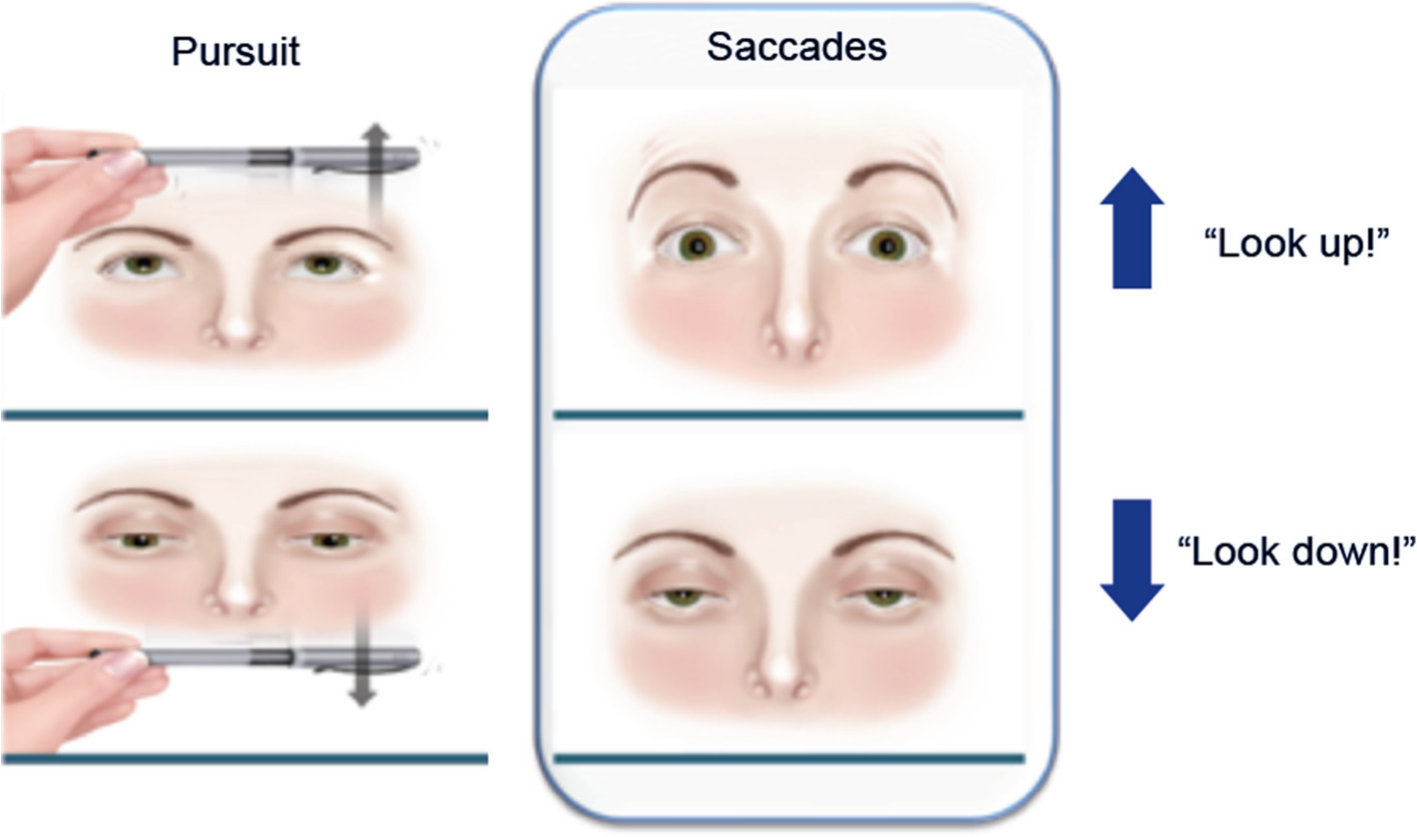
**Clinical assessment of vertical supranuclear gaze palsy.** During the neurological evaluation of eye movements, assessing eye pursuit movements alone (left) is insufficient: voluntary saccades must be tested (right). The examiner should require the subject to visually fixate on two separate objects, e.g. the examiner’s finger and a hatpin, which are displaced first horizontally and then vertically (up and down), but always within the subject’s visual field. The subject is then asked to look at each object alternately.

During the neurological examination, it is important to assess voluntary vertical saccades, and not only eye pursuit movements (Figure 1) [30]. The examiner should require the subject to visually fixate on two separate objects, e.g. the examiner’s finger and a hatpin, which are displaced first horizontally and then vertically, but always within the subject’s visual field. The subject is then asked to look at each object alternately.

VSGP can be misdiagnosed as vertical ocular motor apraxia (OMA), both of which involve impaired vertical saccades. The two can be differentiated by assessing reflexive saccades, which are abnormal in VSGP but more promptly generated in OMA [30]. In addition, VSGP involves reduced saccadic velocity and range, whereas vertical OMA is typically characterised by a deficit in the initiation of voluntary vertical saccades, with normal vertical quick phases of nystagmus [30]. Reflexive saccades can be assessed by asking the patient to look at the examiner’s wiggling finger, which suddenly moves above or below the straight ahead position of the patient [30].

In children, OMA usually occurs in the horizontal plane; therefore, abnormal initiation and speed of vertical saccades makes OMA less likely and favours a VSGP diagnosis.

Other diseases and conditions associated with VSGP include, for example, progressive supranuclear palsy or other tauopathies, multiple system atrophy, dementia with Lewy bodies, spinocerebellar ataxia, Tay-Sachs disease, Wilson disease, vitamin B12 deficiency, Wernicke encephalopathy, Huntington’s disease and Creutzfeldt–Jakob disease [30]. As opposed to these disorders, VSGP development has an earlier onset in NP-C.

#### Ataxia

Ataxia, which is associated with early loss of Purkinje cells in the cerebellum [34,35], is a moderate indicator of NP-C [18]. In combination with dystonic manifestations of the hands and the face it becomes highly suggestive of NP-C.

In NP-C, ataxia presents as ‘slow’ ataxia, manifesting itself in quite slow movements, compared with other ataxic patients. Gait may appear normal in the early stages of the disease. Children with a mild form of NP-C may appear to be slow, for example, walking instead of running, or cautiously taking objects instead of rapidly grabbing them. Ataxia generally appears after dystonia, with the delay between the two symptoms dependent on disease progression, however, in a proportion of infantile and juvenile-onset cases, ataxia may present before dystonia is apparent. In cases where ataxia is the only presenting neurological symptom, it may be useful to wait and watch for additional signs before suspecting NP-C, as ataxia can have multiple aetiologies.

Clinical assessment of ataxia includes provocative tests, such as tandem gait, rapidly alternating movements, finger-to-nose or heel-to-shin tests. Patients with NP-C may be able to adequately perform these tests, but will exhibit slower movements to compensate for their ataxia. Ataxia can be further assessed based on standard scales, such as the International Cooperative Ataxia Rating Scale [36] and the Brief Ataxia Rating Scale [37], which may be more useful in the office setting.

NP-C is part of the expanding group of hereditary autosomal recessive cerebellar ataxias (ARCAs) [38]. However, the following aspects may help distinguish NP-C from other ARCAs: absence of retinal/macular degeneration; lack of peripheral neuropathy in adult patients; marked cerebellar atrophy is usually only observed in advanced stages of the disease; and the presence of VSGP, an almost constant feature which, in combination with splenomegaly, virtually defines the clinical diagnosis of NP-C.

#### Gelastic cataplexy

Gelastic cataplexy, characterised by episodes of sudden loss of muscle tone that can cause the patient to collapse or fall, is one of the strongest neurological indicators of NP-C [18]. Although relatively rare, it is a strong predictor of NP-C when it occurs in combination with other manifestations, such as VSGP. Gelastic cataplexy is not associated with loss of consciousness, abnormal vigilance or altered awareness. In NP-C, gelastic cataplexy presents along a continuum, from rubbery feeling in the legs and minor head-drops to full collapse of the entire body [1], and can appear as early as 2 years of age.

A history of drop attacks or loss of posture associated with emotional stimuli (e.g. laughing or crying) should raise the suspicion for gelastic cataplexy. This sign may be missed in children, as falls due to cataplexy may be often misinterpreted as secondary to cerebellar ataxia [1,39]. Gelastic cataplexy, characterised by a normal electroencephalogram (EEG), must be differentiated from gelastic seizures, which exhibit clinical features of epilepsy and usually abnormal EEG. As gelastic cataplexy can be part of the narcoleptic tetrad, which includes cataplexy, narcolepsy, sleep paralysis and hypnagogic hallucinations, it is important to exclude narcolepsy, which is not observed in patients with NP-C.

#### Dystonia

Dystonia is a neurological movement disorder characterised by excessive involuntary muscle contraction as a result of pathology in the basal ganglia and, to a lesser extent, the cerebellum [35,40]. This symptom is very common and, if present in patients with NP-C, occurs more frequently in the adolescent/adult onset form than in the juvenile form [2,39] and is a moderate indicator of the disease [18].

In NP-C, dystonia rarely presents in isolation but usually with ataxia, a combination highly specific for NP-C [17]. Occasionally, dystonia may present in isolation of ataxia, which can lead to misdiagnosis as genetic forms of primary dystonia, for example DYT1 and DYT6, In NP-C, dystonia affects the extremities and the face. During later stages of the disease, dystonia may also involve the neck and trunk, and as illness progresses it can also affect gait [41]. Typical dystonic features in NP-C include focal hand dystonia with wrist flexion, a forced (subtle) smile when speaking, resulting from dystonia of the jaw musculature and wrinkles on the forehead. Dystonia tends to worsen during intercurrent illnesses. Differential diagnosis of dystonia in inborn errors of metabolism include respiratory chain disorders, pyruvate dehydrogenase deficiency, glucose transporter 1 deficiency, vitamin E deficiency, organic acidaemia, urea cycle disorders, homocystinuria and Wilson disease. Genetic forms of primary dystonia, such as DYT1 and DYT6, are not strictly neurodegenerative.

#### Dysarthria/dysphagia

Dysarthria results in slurred and irregular speech with impaired pronunciation, due to a lack of coordination of the motor-speech system [42]. It results from a combination of ataxia and dystonia and involves pathologies in the cerebellum and basal ganglia. Dysarthria is a moderate indicator of NP-C [18] which, in combination with other symptoms, increases the diagnostic likelihood of NP-C.

Dysphagia, or difficulty in swallowing, is associated with dysfunction not only in the brainstem (affecting motor and sensory functions of swallowing), but also in cortical areas in the frontal lobe (responsible for swallowing initi-ation as well as management and retention of safe swallowing strategies) [43,44]. This common symptom of NP-C may appear early or later in the disease course [1]. It is a moderate indicator of NP-C and its combination with other symptoms is specific to the disease [18]. Dysphagia represents a major problem as it correlates with aspiration pneumonia, one the most common causes of death of NP-C [45,46]. Swallowing function can be assessed by standardised swallowing assessments of different substances, and via investigations such as video fluoroscopy and fiberoptic endoscopic evaluation of swallowing [47-49].

#### Hypotonia

Hypotonia, the first neurological signs of NP-C appearing in the second year of life, is a non-specific indicator of the disease, but early onset of hypotonia around or before the first birthday is associated with the more progressive infantile type of NP-C [18]. In toddlers, clumsiness results from a combination of hypotonia, beginning of ataxia and abnormal eye movements, which lead to stumbling over obstacles. In school children clumsiness is usually related to ataxia and may manifest as the deterioration of handwriting. This deterioration is related to the onset of dysmetria, a lack of movement coordination that can be detected using the Archimedes spiral test.

#### Delayed developmental milestones

Delays in the developmental milestones are an ancillary indicator of NP-C [18]. These include motor delays (e.g. slow movements while walking and transferring objects, clumsiness, poor head control), speech delay, vision/ocular-motor developmental delay, and social delay (e.g. interactive play). When associated with other manifestations such as isolated splenomegaly without hepatomegaly and VSGP, this sign becomes specific to NP-C. Virtually all children with NP-C exhibiting developmental delays have a history of splenomegaly. In practice, the range and extent of developmental delay can vary widely between patients, but can be very useful for early diagnosis in infant and juvenile patients. The majority of infants will typically fail to reach some, or all, of the following developmental milestones, in the following chronological order: delays in picking up and transferring small objects; lack of visual attention; delayed walking; frequent falls and a tendency cruise/hold onto parents/solid objects to aid balance. A common feature in juveniles is a delay in language, until 3–4 years old. However, this sign is not specific to NP-C as speech delay may have many causes, and other signs and symptoms should therefore be checked.

#### Seizures

Seizures are not specific to NP-C and are considered an ancillary indicator of the disease [18]. In NP-C, seizures can be partial/focal, or generalised, myoclonic or tonic-clonic, and can vary substantially in frequency and intensity [1]. In the differential diagnosis process, it is important to exclude other disease involving seizures, e.g. myoclonic epilepsy or mitochondrial disease.

#### Hearing loss

High frequency sensorineural hearing loss has been reported in NP-C and can be light or severe [2,5]. It affects about 20% of the patients and appears to be more frequent in adults. Hearing ability can be tested by audiograms or auditory brainstem responses.

It is believed that cholesterol, whose trafficking is impaired in NP-C, plays a key role in auditory physiology [50,51]. Animal studies have shown that the cholesterol-chelating agent 2-hydroxypropyl-β-cyclodextrin, a promising experimental therapy for NP-C, may have deleterious effects on hearing impairment [52,53], emphasising the need for auditory testing in patients receiving this treatment.

### Psychiatric symptoms in NP-C

#### Cognitive decline

Progressive cognitive decline affects all patients with NP-C and is a strong indicator of the disease [18]. In combination with VSGP, cognitive decline is strongly suggestive of NP-C.

In adult forms of NP-C, the typical profile is characterised by initial deficits in executive functioning, followed by memory impairment and cognitive slowing. In adult patients initial changes may be subtle. Executive impairment includes very early disinhibition, perseveration (i.e. inflexibility in thinking and inability to shift set), poor judgement, lack of insight, impaired ability for abstraction, attentional deficits, and cognitive slowing. Disinhibition is often the earliest sign, detected neuropsychologically by tools such as the Stroop colour-word test [28]. However, memory impairment can also manifest as an early feature.

Cognitive impairment in children manifests as a delay in normal cognitive development, or mental retardation. It is common that children reach a certain stage of development, stop progressing, and start showing cognitive decline associated with functional loss. Attention-deficit hyperactivity disorder may be present in childhood as a precursor to adolescent or adult development of NP-C.

Memory deficits, including impaired formation of new memories and disorientation, reflect hippocampal abnormalities [54,55]. Cognitive slowing may be due to changes in white matter causing disconnection of frontal-subcortical circuitry [56]. In addition, direct striatal pathology causes cognitive slowing through disruption of frontostriatal loops [57].

Cognitive impairment is commonly screened for by performing a Mini-Mental State Evaluation [58]. Although not sensitive in early stages of NP-C, it is useful for assessing cognitive function in patients with moderately severe NP-C in more advanced stages. Other traditional neuropsychological tests for global cognition and memory include the Wechsler Adult Intelligence Scale (WAIS) [59] and the Wechsler Memory Scale (WMS). Tests for executive dysfunction include the trail-making test and the Controlled Oral Word Association test [60]. Moreover, memory deficits can be assessed, for example, by the Rey Auditory Verbal Learning Test (RAVTL) [61].

In children, cognitive impairment may be monitored using the Bayley Scales for Infant Development [62], Vineland Adaptive Behaviour Scales [63], the Wechsler Intelligence Scale for Children [64], the Denver Developmental Screening Test [65] and the Griffiths Mental Development Scale [66].

NP-C should be differentiated from Alzheimer’s disease (AD), also characterised by cognitive decline and short-term memory loss. Cognitive impairment in NP-C is characterised by a more pronounced prefrontal involvement, as opposed to the more generalised dementia and different degree of cognitive slowing observed in patients with AD. As a result, patients with NP-C mostly exhibit deficits in executive function and disinhibition. Whilst executive deficits in adult patients in NP-C are similar to those observed in patients with frontotemporal dementia [67], the presence of VSGP and motor signs such as ataxia and dystonia allows readily differentiating the two diseases.

#### Psychosis

Psychosis is characterised by hallucinations, delusions and/or thought disorder and is a moderate indicator of NP-C [18]. In NP-C, psychotic symptoms typically present in adolescence or early adulthood, may be treatment resistant and sensitive to neuroleptic side effects (particularly dystonia), and may be associated with secondary signs (visual hallucinations, catatonia and fluctuating symptoms) [68].

#### Disruptive or aggressive behaviour in adolescence and childhood

Disruptive or aggressive behaviour in adolescence and childhood is an ancillary indicator of NP-C [18]. In pubescent patients, it may present in addition to cognitive impairment and behavioural disinhibition.

### Diagnosis and diagnostic methods

As described earlier, NP-C presents in a highly heterogeneous manner, sometimes with atypical phenotypes, which makes the disease difficult to detect.

An NP-C Suspicion Index (SI) tool was recently developed to aid clinicians identify patients with suspicion of NP-C, for whom other common diseases have been ruled out [18]. This highly specific, sensitive, easy-to-use and reliable tool provides information about symptomatology and presentation patterns in NP-C [18].

The diagnostic process includes recording a full medical history and a comprehensive clinical and neurological examination to detect characteristic signs and symptoms, followed by a differential diagnostic procedure to exclude other possible causes and, finally, confirmation of NP-C diagnosis by biochemical (filipin staining) and genetic testing [1,69-71]. As part of genetic counselling, heterozygous carriers should always undergo genetic testing in order to reliably determine their genetic status, which is useful for family planning implications [1]. Oxysterols (cholesterol oxidation products) have recently been shown to be significantly elevated in the plasma of patients with NP-C1 [72,73], thus bearing the potential for being used as biomarkers for NP-C. Measurement of plasma oxysterol levels is recommended as a supplementary test for cases with unclear NP-C genetic mutations and biochemical phenotypes [1].

### Disease management and treatment

Detailed guidelines have been recently published on disease management including treatment [1]. In the absence of a curative treatment, improving or maintaining patients’ quality of life and their neurological and mental functions is considered the best possible reasonable goal. Optimal disease management should rely on a multidisciplinary treatment approach, combining symptomatic treatment, close community support and disease-specific therapy. To date, miglustat is the only disease-specific approved therapy for the treatment of progressive neurological manifestations in paediatric and adult patients with NP-C [7]. Miglustat has been shown to improve or stabilise key parameters of neurological disease progression in children, and in juvenile and adult patients, both in clinical trials and in clinical practice settings [47,49,74-79].

In order to stabilise or slow the progression of irreversible neurological damage, disease-specific therapy with miglustat should be initiated at the earliest signs of neurological manifestations [1].

### Prognosis

NP-C is a progressive, irreversible and chronically debilitating disease leading to premature death, usually between the ages of 10 and 25 years according to previous studies [70]. However, this figure may no longer be valid as more late-onset patients are now diagnosed in adulthood. Prognosis largely correlates with age at onset of neurological signs, whereby early-onset forms progress faster [1,2].

Dysphagia has been identified as a major risk factor for mortality in patients with NP-C [46]. In fact, impaired swallowing is associated with aspiration pneumonia, the most common cause of death in neurodegenerative disease including NP-C [45,46,80]. Improving swallowing function may therefore help increase patients’ life expectancy.

### Unresolved questions and future perspectives

Despite increasing research in the field, open questions remain regarding the exact function of NPC1 and NPC2 proteins, as well as the precise role of sphingosine and other lipids in the pathogenesis of NP-C [14]. As such, further investigations are required to elucidate the biochemical and cellular mechanisms leading to the disease, in order to design new targeted therapies. Moreover, this may help understand the connection between a traffic lipid disorder and the various neurological phenotypes observed in patients.

The lack of a single, definitive diagnostic test for all populations means that NP-C remains largely under- or misdiagnosed. Currently available screening and diagnostic techniques are not straightforward and may contribute to delays in diagnosis. The advent of faster and cheaper genetic tests such as Next-Generation Sequencing, as well as the potential use of plasma oxysterols as biomarkers of NP-C [1,72,73], will likely have a great impact on future screening and diagnostic strategies for NP-C and other rare diseases. Establishment of a test to implement oxysterols for their potential use as screening and diagnostic biomarkers is currently under development. Furthermore, preliminary data indicate that levels of certain oxysterol species correlate with disease severity in patients with NP-C1 [73]. However, further data are required to determine whether oxysterols may be used as biomarkers for monitoring disease progression.

Walterfang et al. have recently shown that patients with NP-C exhibit alterations in brain morphology [35,81-83]. Preliminary data suggest that miglustat can maintain brain volume in treated patients compared with untreated control subjects. However, further evidence is required to establish whether miglustat has an effect on brain morphology and whether magnetic resonance imaging measures can be used to monitor disease progression (Walterfang, unpublished data).

## Conclusions

Detection of NP-C remains challenging, due to the highly heterogeneous presentation of the disease, with manifestations occurring along a continuum. While individual signs and symptoms may not be specific to NP-C, their specific combinations can be an important indication of the disease [18]. Therefore, single findings are often insufficient and further investigations to identify any other symptom should always be performed when a diagnosis is lacking.

Correct assessment of different symptoms is crucial to identifying the disease. A combination of splenomegaly, VSGP and cognitive impairment, together with other, non-specific visceral, neurological or psychiatric manifestations, is highly predictive of NP-C. Tools such as the NP-C Suspicion Index tool may offer considerable help in the diagnostic process [18].

Minimising the delay in diagnosis is crucial for prompt initiation of disease-specific therapy in order to slow progression of neurological disease and to improve patient care and treatment outcomes. This expert-based clinical description of NP-C signs and symptoms is a further step towards raising disease awareness and improving early detection, a goal for which the medical community strives in the near future.

## Abbreviations

AD: Alzheimer’s disease; ARCAs: Autosomal recessive cerebellar ataxias; EEG: Electroencephalogram; GD3: Gaucher disease type 3; NP-C: Niemann-Pick disease type C; NP-C1: Niemann-Pick disease type C caused by mutations in the *NPC1* gene; NP-C2: Niemann-Pick disease type C caused by mutations in the *NPC2* gene; OMA: Ocular motor apraxia; VSGP: Vertical.

## Competing interests

EM, HHK, CML, CJH, FS, MW have all received consulting fees or honoraria from Actelion Pharmaceuticals Ltd, Allschwil, Switzerland. SAK is an employee of Actelion Pharmaceuticals Ltd, Allschwil, Switzerland.

## Authors’ contributions

All authors were involved in the preparation of the draft manuscript and have read, critically reviewed and approved the final version of the manuscript.
